# Emergency Etoposide-Cisplatin (Em-EP) for patients with germ cell tumours (GCT) and trophoblastic neoplasia (TN)

**DOI:** 10.1186/s12885-019-5968-7

**Published:** 2019-08-05

**Authors:** Charleen Chan Wah Hak, Christopher Coyle, Arwa Kocache, Dee Short, Naveed Sarwar, Michael J. Seckl, Michael A. Gonzalez

**Affiliations:** Department of Medical Oncology, Imperial College Healthcare National Health Service Trust, Charing Cross Hospital, Fulham Palace Road, London, W6 8RF UK

**Keywords:** Germ cell tumour, Trophoblastic neoplasia, Emergency chemotherapy, Service delivery, Teenagers and young adults

## Abstract

**Background:**

Etoposide (E) at 100 mg/m^2^ combined with Cisplatin (P) at 20 mg/m^2^ represents an induction 2-day regimen embedded in our clinical practice for patients with advanced GCT or TN at high risk of early death. We evaluated 24/7 Em-EP administration to a combined GCT-TN cohort at our Emergency Cancer Treatment Centre (ECTC) to determine its efficacy within the acute setting.

**Methods:**

Patients who received Em-EP during a five-year interval were identified from electronic databases at Imperial College Healthcare NHS Trust. Data collected included demographics, treatment details and clinical outcome.

**Results:**

Em-EP was administered in the emergency setting to 104 patients, predominantly young adults (median age 35, range 17–71). Half the cases were GCT (*n* = 52): 22 male (6 seminomas, 13 non-seminomas); 30 female (2 dysgerminomas, 28 non-dysgerminomas). The other 50% were treated for TN (*n* = 52): 45 gestational (GTN) and 7 non-gestational. Most patients received Em-EP for a new cancer diagnosis (*n* = 100, 96%), within 24 h (*n* = 93, 89%) and out-of-hours (*n* = 74, 70%). Indications for Em-EP included symptomatic disease (*n =* 66, 63%), high-burden disease, (*n =* 51, 49%) and organ failure requiring Intensive Care Unit support (*n =* 9, 9%). Neutropenic sepsis was observed in 5%. Four-week overall survival after Em-EP administration was 98%.

**Conclusions:**

Despite the potentially fatal complications encountered in the acute setting, early mortality with Em-EP is low at our ECTC. Specialist units that treat unwell patients with advanced GCT or TN should consider making Em-EP available 24/7 for emergency administration. Its efficacy within a prospective cohort and in other platinum-sensitive malignancies requires evaluation.

**Electronic supplementary material:**

The online version of this article (10.1186/s12885-019-5968-7) contains supplementary material, which is available to authorized users.

## Background

Germ cell tumours (GCT) and trophoblastic neoplasia (TN) are highly chemosensitive diseases that can present with life-threatening complications in teenagers and young adults [21, 22]. An advanced stage at presentation further compromises the favourable clinical outcome that is frequently associated with these disease types when diagnosed at an early stage [21, 22]. GCT can arise from testicular, ovarian or extragonadal primary sites and bulky abdominal lymphadenopathy or extensive lung metastases in advanced disease can cause profound renal impairment or acute respiratory distress, respectively. Patients with intracranial metastases also harbour a less favourable prognosis and can present symptomatically [8]. Analogously, TN encompasses a range of highly vascular diseases that include hydatidiform moles and choriocarcinomas. TN patients with advanced disease are at high risk of early death from fatal haemorrhage [13, 14].

In addition to the serious disease-related complications, organ failure in patients presenting with advanced GCT and TN can compromise their ability to receive conventional chemotherapy upfront. Standard regimens for GCT include BEP (Bleomycin, Etoposide and Cisplatin) [20], EP (Etoposide and Cisplatin) [3] or a dose-intense combination such as POMB-ACE (Cisplatin, Vincristine, Methotrexate, Bleomycin, Actinomycin D, Cyclophosphamide and Etoposide) [2]. In patients with high-risk TN, EMA-CO (Etoposide, Methotrexate, Actinomycin D, Cyclophosphamide and Vincristine) is often the management standard [6, 12, 15]. Due to the associated toxicities, any of these regimens at full dose would be considered unsuitable for immediate administration to patients presenting with symptomatic or high-burden GCT or TN, particularly when diminished reserves from organ failure prevail or when there is a co-existing poor performance status at baseline.

Here, we describe a 5-year experience with the intravenous Emergency Etoposide-Cisplatin (Em-EP) regimen that is embedded in our routine clinical practice. In Em-EP, Etoposide (E) at 100 mg/m^2^ combined with Cisplatin (P) at 20 mg/m^2^ is administered on Day 1 and Day 2, although single or 3 consecutive daily alternatives may also be indicated. We reserve Em-EP for unwell GCT or TN patients presenting acutely to our Emergency Cancer Treatment Centre (ECTC) based at Charing Cross Hospital, available 24 h a day, 7 days per week (24/7). Following initial Em-EP administration, patients proceed onto standard full-dose chemotherapy when deemed fit. If still clinically unwell or compromised by organ failure, further Em-EP cycles can be administered on a weekly basis prior to embarking on standard chemotherapy. This study represents the first analysis on emergency chemotherapy administration for a combined GCT and TN patient cohort. Service delivery and adherence to Institutional guidelines [9, 10] were evaluated, in addition to clinical outcome data including early survival.

## Methods

### Patients

**Fig. 1:**
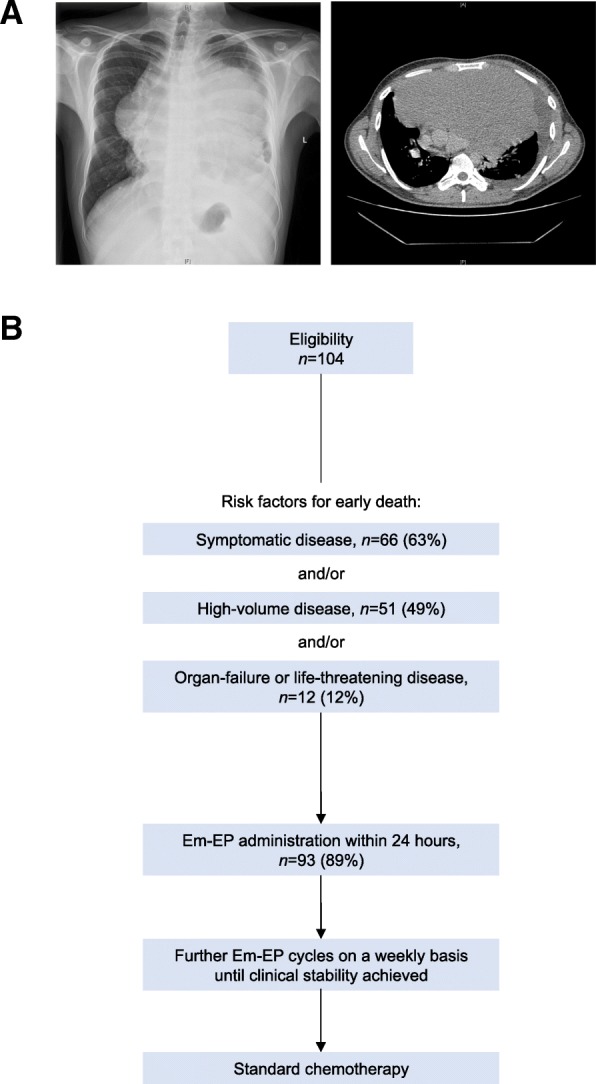
Em-EP Study details. **a** Primary mediastinal seminoma in a young adult presenting with acute respiratory distress and treated urgently with Em-EP within 24 h following admission. Chest radiograph and computed tomography identified a large anterior mediastinal mass measuring 20.7 cm × 13.3 cm, occupying nearly the entire thorax and compressing the great vessels and bronchi. **b** Study design. Retrospective evaluation for patients with advanced GCT and TN admitted to our ECTC for Em-EP

Imperial College Healthcare NHS Trust serves as a National tertiary referral centre for GCT and TN. Unwell cancer patients are admitted through a 24/7 Emergency Cancer Treatment Centre (ECTC) based at Charing Cross Hospital. In this study, we retrospectively evaluated all patients who received Em-EP between 1st January 2012 and 31st December 2016 (Fig. 1). In our clinical experience, symptomatic male GCT patients in an intermediate or poor IGCCCG prognostic group [11] and high-risk female GTN patients with an International Federation of Gynecology and Obstetrics (FIGO) score ≥ 7 are frequent recipients [1]. Low-risk GTN patients with a FIGO score < 7 who present acutely with extremely high hCG values of > 400 000 IU/L usually acquire resistance to single-agent Methotrexate and are treated with combination chemotherapy (e.g. EMA-CO) from the outset, as for high-risk patients [17]. This patient group warrants initial treatment with Em-EP when standard combination chemotherapy is unavailable out-of-hours.

Patients are treated as GCT if they have either a histologically-confirmed diagnosis or elevated serum tumour markers with a co-existing gonadal mass. TN patients are classified here as gestational TN (GTN) and non-gestational TN. For the purposes of this study, female patients without an antecedent pregnancy are classified as non-gestational TN if they presented with a probable advanced trophoblastic malignancy based on an elevated hCG level with or without a uterine mass. Patients diagnosed clinically with an AFP and/or hCG-secreting malignancy and no obvious primary site were also treated empirically but excluded from survival analysis.

Data was collected between May and August 2017 from electronic patient records, chemotherapy databases and hand-written patient notes. Data collected included patient demographics, cancer staging, treatment, resistance to therapy, disease relapse and survival information. Symptomatic disease was identified if documented in clinical records, for example, uterine or vaginal bleeding, abdominal pain or dyspnoea. High-burden disease was defined as multiple disease sites or a single disease site measuring > 5 cm. Patients were classified as having organ failure or life-threatening disease if they required High Dependency Unit (HDU) or Intensive Care Unit (ICU) admission for support. Scoring systems used included the FIGO prognostic and staging score for TN, FIGO staging for malignant GCT in females and IGCCCG prognostic classification and Union for International Cancer Control (UICC) staging for GCT in males. Local ethics and information governance approval was obtained.

### Emergency chemotherapy

Em-EP is currently the only chemotherapy regime to be reconstituted out-of-hours at our Institution. Out-of-hours is defined as 8 pm to 8 am on weekdays from Monday to Friday and all hours at the weekend on Saturday and Sunday. Clinical parameters screened by pharmacy include the body surface area and a calculated Cockroft-Gault creatinine clearance for Cisplatin based on an ideal body weight. To anticipate emergency treatment required at weekends, the Aseptic Chemotherapy Unit prepares Em-EP in advance for 2 patients at a dose based on a 2 m^2^ body surface area. Em-EP can be administered over 1, 2 or 3 consecutive days. Cycles can be repeated every 7 days depending on the clinical response, with a subsequent change in regimen once the clinical condition has improved with resolution of any organ dysfunction.

## Results

### Patients treated with Em-EP

Between 1st January 2012 and 31st December 2016, Em-EP was administered to 104 patients in the acute setting, predominantly to young adults with a median age 35 (range 17–71). Hence, approximately 20 young adults per annum with advanced GCT or TN receive emergency chemotherapy at our Centre. We observed an equal split within the cohort for patients requiring Em-EP, with half the cases diagnosed with GCT (*n =* 52) and the other half with TN (*n =* 52) (Table 1). Intra-uterine (*n* = 44, 42%) and gonadal (*n* = 41, 39%) primary sites were the most common. The majority of patients received Em-EP at their initial diagnosis (*n* = 100, 96%) rather than for recurrent disease (n = 4, 4%). Indications for treatment with Em-EP included symptomatic disease in 63% (*n* = 66), high-burden disease in 49% (*n* = 51) and organ failure or life-threatening disease in 12% (*n =* 12). Hence, patients identified within our study cohort are presenting late with advanced disease in the acute setting, thereby requiring emergency treatment. The median follow-up period at the time of analysis was 9 months (range 0–64 months).

**Table 1:** Patient characteristics for GCT-TN cohort within the Em-EP study

Total number of patients, *n* (%)	*n* = 104	
Age, years	Median: 35 Mean: 36 Range: 17–71	
Gender		
Male	22^a^ (21%)	
Female	82^b^ (79%)	
Primary site of disease		
Gonadal, *n* = 41 (39%)		
Testicular, *n* = 15 (14%)	Seminoma	4 (4%)
	Non-seminomatous GCT	11 (11%)
Ovarian, *n* = 26 (25%)	Dysgerminoma	2 (2%)
	Non-dysgerminomatous GCT^c^	24 (23%)
Uterine, *n* = 44 (42%)	Gestational trophoblastic neoplasia (GTN)	
	Hydatidiform mole	19 (18%)
	Choriocarcinoma	24 (23%)
	Non-gestational choriocarcinoma	1 (1%)
Extra-gonadal, *n* = 8 (8%)		
Mediastinal		5 (5%)
Retroperitoneal		1 (1%)
Sacrococcygeal		1 (1%)
Pineal		1 (1%)
Other, n = 2 (2%)		
Pulmonary^d^		2 (2%)
Unknown primary, *n* = 9 (8%)		
GCT		2 (2%)
TN		6 (6%)
Adenocarcinoma of unknown origin^e^		1 (1%)

^a^ Six male patients harboured seminomas (32%) and 13 non-seminomatous GCTs (68%). In the remaining 3/22 male patients (14%), 1 patient (5%) presented with both a raised serum hCG and a testicular mass, whereas 2 patients (9%) were treated as GCT based on elevated serum tumour markers alone. Primary disease sites for male patients included testicular in 68% (n = 15), extragonadal in 23% (mediastinal, retroperitoneal and pineal; n = 5) and unknown in 9% (n = 2)

^b^ In contrast to their male counterparts, a histological diagnosis was available for all 30 female GCT patients admitted in the acute setting. Subtypes included 2 dysgerminomas (7%) and 28 non-dysgerminomas (97%). This subset excludes non-gestational trophoblastic neoplasia. Primary disease sites were ovarian in 87% (*n* = 26) and extragonadal in 10% (mediastinal, sacrococcygeal, pulmonary; n = 3). A primary site was unknown in 1 patient (3%)

^c^ One patient with an ovarian primary disease site was diagnosed as an ectopic gestational choriocarcinoma

^d^ Histology: yolk sac tumour; non-gestational epithelioid trophoblastic tumour (excluded from survival analysis)

^e^ Empirical treatment as for GCT due to a high AFP level (excluded from survival analysis)

### Germ cell tumour (GCT) patients

**Fig. 2:**
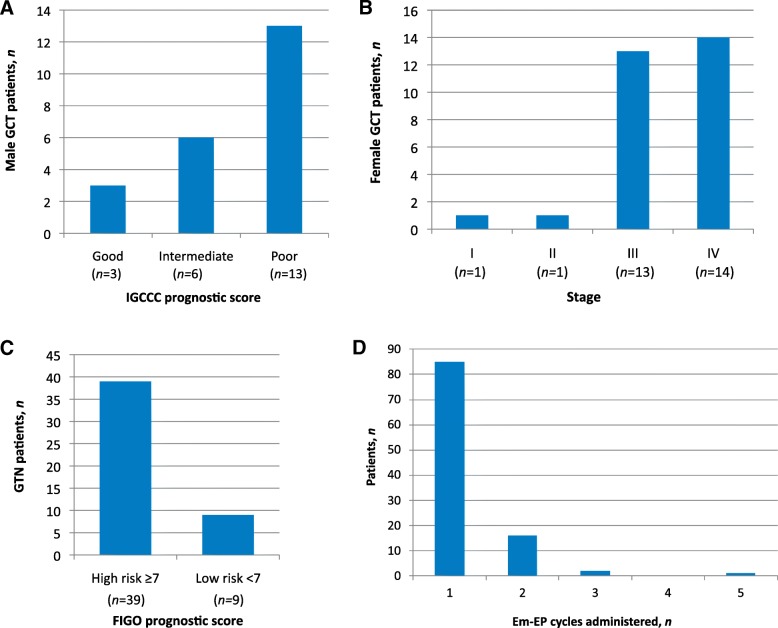
Patient characteristics and Em-EP delivery. **a** Male GCT patients who received Em-EP (*n* = 22) mostly presented with IGCCCG intermediate or poor-risk disease. 19/22 male patients (86%) were treated for disease confirmed histologically. Metastatic sites for patients with gonadal primaries (*n* = 15) included pulmonary only (*n* = 5, 33%) and non-pulmonary visceral disease (liver, brain and presumed peritoneal disease with ascites; n = 5, 33%). No visceral metastases were present in 5 males (33%) who had symptomatic high-volume lymphadenopathy, which in 2 cases led to bilateral hydronephrosis and renal dysfunction. **b** Female GCT patients (*n* = 29) frequently received Em-EP for advanced stage disease. Metastatic sites included pulmonary only (*n* = 6, 20%) and non-pulmonary visceral disease (liver, brain, bone, bowel and abdominal wall; *n* = 10, 33%). 13 female patients were free from visceral metastases at their emergency presentation (43%) but presented symptomatically from high-volume primary disease, bulky lymphadenopathy or malignant ascites. **c** FIGO prognostic score for GTN patients (*n* = 45). Metastatic sites included pulmonary only (n = 22) and non-pulmonary visceral disease (central nervous system, kidneys, liver, bladder, spleen, colon and thyroid) (*n* = 8). 15 GTN patients (33%) had no visceral metastases at presentation. **d** Frequency distribution for Em-EP cycles administered. Multiple weekly cycles were used in patients deemed too unwell, for example due to organ failure, before standard full-dose chemotherapy with either: 2 cycles (*n* = 16, 15%), 3 cycles (*n* = 2, 2%), 4 cycles (*n* = 0, 0.0%) or 5 cycles (n = 1, 1%)

In the acute setting, 22/52 GCT patients treated with Em-EP were male (42%) with a median age 30 (range 20–71). 30/52 GCT patients were female (58%) and most (*n* = 22, 73%) were also young adults with a median age 32 (range 21–69). Non-seminomatous GCTs in male patients, the equivalent non-dysgerminomas in female patients and GCT patients of either sex with a gonadal primary represent the majority of cases receiving Em-EP within our study cohort. Most male patients (*n* = 22, 81.2%) presented as an emergency with IGCCCG intermediate or poor-risk disease (Fig. 2a). In male GCT patients with gonadal primaries, most presented acutely with Stage III disease: Stage I (*n* = 0), Stage II (*n* = 3, 20%) and Stage III (*n* = 12, 80%). Female GCT patients requiring Em-EP also presented with advanced disease, with 90% who received Em-EP having been diagnosed with Stage III or IV disease (*n* = 27) (Fig. 2b). The average pre-treatment serum hCG and LDH levels were higher in male GCT patients receiving Em-EP (hCG 92 701 IU/L, LDH 791 IU/L) than in females (hCG 1 746 IU/L, LDH 706 IU/L). Conversely, the average pre-treatment AFP was higher in female GCT patients (17 389 ng/ml) than in males (5 928 ng/ml).

### Trophoblastic neoplasia (TN) patients

In our cohort, 52 female patients requiring Em-EP presented with TN, of which 45 (87%) presented with gestational trophoblastic neoplasia (GTN). As for our GCT patients, most TN patients were young adults with a median age 36 (range 17–57). For the 45 GTN patients, disease subtypes included hydatidiform mole (*n =* 19, 42%) and choriocarcinoma (*n =* 26, 58%). Forty-one patients (91%) were defined as high-risk with a FIGO prognostic score ≥ 7 and 4 (9%) were classified as low-risk (FIGO prognostic score < 7) (Fig. 2c). All 4 patients classified as low-risk patients (FIGO prognostic score = 6) had hCG levels > 400 0000 IU/L and deemed likely to develop resistance to single-agent chemotherapy [1]. Two of these patients presented out-of-hours and received initial treatment with Em-EP when standard combination chemotherapy (EMA-CO) was unavailable. Moreover, 3 of these 4 patients had rising or plateaued hCG levels after an evacuation of retained products of conception (ERPC) (*n =* 3, 75%) and one was symptomatic with ongoing vaginal bleeding that required arterial embolisation (*n* = 1, 25%). FIGO stage distribution was as follows: Stage I (*n* = 13*,* 27%), Stage II (*n* = 2*,* 42%), Stage III (*n* = 21*,* 44%), Stage IV (*n* = 9, 25%). Indications for Em-EP included a high FIGO score, continuous vaginal bleeding and rising or plateaued hCG levels post-ERPC. Seven patients with non-gestational trophoblastic neoplasia presented in the acute setting with uterine (*n* = 1, 14%), lung (n = 1, 14%) and unknown (*n* = 5, 71%) primary sites.

Pre-treatment serum hCG was highly elevated in TN patients at their emergency presentation with an average value at 518 394 IU/L. No significant change was observed when comparing this value to the average hCG measured after the first Em-EP administration (*p* = 0.08, paired two-tailed t-test).

### Service delivery for emergency chemotherapy

Most patients (*n* = 74, 70%) received emergency chemotherapy out-of-hours. Em-EP was administered to 64 patients during a weekday between 8 pm and 8 am (61%) and to 19 patients at the weekend (18%). 93/104 patients (*n* = 93*,* 89%) received Em-EP within 24 h from their emergency presentation (mean 0.88 days, median 0 days; Additional files 1 and 2). Amongst 9 patients admitted to the Intensive Care Unit (ICU) during their initial inpatient stay, 5 patients (56%) received their first Em-EP cycle on ICU within 24 h from presentation. Patients were admitted to ICU for respiratory support (*n* = 3, 33%), inotropic support (*n* = 1, 11%), both respiratory and inotropic support (*n* = 2, 22%) and monitoring only (*n* = 3, 33%). Three patients required HDU admission for respiratory support (*n* = 2, 67%) or monitoring only (*n* = 1, 33%).

Twenty-one (95%) male GCT patients, 29 female GCT patients (97%) and 50 TN patients (96%) received Em-EP at our ECTC as first-line treatment. One male GCT patient (5%), 1 female GCT patient (3%) and 2 TN patients (4%) had previously received standard chemotherapy and presented to our ECTC with disease relapse. Although Em-EP can be administered over 1, 2 or 3 consecutive days [4, 17], all patients in our cohort received the 2-day Em-EP regimen with repeated cycles administered according to clinical response and a switch to standard chemotherapy once their condition had improved. Most patients (*n* = 85, 82%) required only one Em-EP cycle following admission (Fig. 2d) and all patients were admitted to the ECTC for their first Em-EP cycle. Since 2014, annual Em-EP use within the emergency setting has steadily been increasing: 2012 (*n =* 21), 2013 (*n =* 13), 2014 (*n =* 20), 2015 (*n =* 21) and 2016 (*n =* 29). The average inpatient stay was 19 days (median 10 days, range 1–262 days).

### Neutropenic complications with Em-EP

Following admission and prompt treatment with Em-EP, 9 patients (9%) developed neutropenic sepsis and 13 patients (13%) developed non-neutropenic pyrexia. Amongst the 9 patients who developed neutropenic sepsis, 5 (5%) occurred post Em-EP and prior to the first cycle of standard chemotherapy. Three of these 5 patients were then started on Granulocyte Colony Stimulating Factor (G-CSF). 4/9 neutropenic events were unrelated to the initial Em-EP and occurred after starting standard chemotherapy with a permissible full blood count at baseline.

### Clinical outcome following Em-EP and standard chemotherapy

Within the entire cohort, 102 patients (98%) remained alive at 4 weeks after their first Em-EP administration, with only 2 early deaths (2%) observed at less than 4 weeks after Em-EP (Table 2). Therefore, with full escalation and full support, Em-EP can be a life-saving intervention in patients with advanced GCT and TN who present with life-threatening disease.

**Table 2:** Clinical outcome for bona fide GCT and GTN patients who received Em-EP. Median follow-up time, 9 months

Clinical outcome	GCT patients, *n = 50* (%)	GTN patients, *n* = 45 (%)
Alive at 4 weeks	49 (98%)	44 (98%)
Alive at 6 months	46 (92%)	44 (98%)
Resistant disease (first relapse)	19 (38%)	8 (18%)
Alive at follow-up	36 (72%)	44 (98%)
Deaths at follow-up	14 (28%)	1 (2%)
Causes of death	• Disease progression (n = 8, 57%) • Sepsis (n = 2, 14%) • Unknown (n = 4, 29%)	• Disease progression (n = 1, 100%)

The 2 patients who died within 4 weeks included 1 male GCT patient and 1 non-gestational TN patient. A 45 year-old gentleman of no fixed abode presented at our ECTC with an advanced seminoma that had arisen within an undescended right-sided pelvic testis resulting in right hydronephrosis. Right para-aortic lymphadenopathy was also present. He sadly died elsewhere from an unknown cause on Day 25, having received Em-EP on Day 4 following admission and having initiated BEP chemotherapy on Day 9. The second patient died on Day 11 at a Respiratory ICU, transferred from our own ICU for extracorporeal membrane oxygenation. She was a 40 year-old lady admitted with septic shock and respiratory failure from a symptomatic right-sided pleural effusion that was exudative and had required multiple intercostal drains for prompt symptomatic relief, as well as a video-assisted thoracoscopic surgical pleurodesis that had proved unsuccessful. Pleural fluid analysis had identified both *Staphylococcus aureus* and extended-spectrum beta-lactamase-producing bacteria. The baseline serum HCG was elevated at 4772 IU/L. Em-EP had been administered within 24 h of admission, whilst intubated, ventilated and on both inotropes and intravenous antibiotics.

In our total patient cohort with a minimum 6-month follow-up, overall survival was 81% with 20 patient deaths. Specifically, there were 15 deaths in the cohort of 95 patients (45 GTN, 20 male GCT, 30 female GCT) excluding non-gestational TN patients, patients with cancer of unknown primary without confirmed histology, poorly-differentiated tumour marker-secreting tumours or lung cancer treated empirically as GCT or GTN (Table 2). Overall survival was higher in bona fide GTN (98%) compared to bona fide GCT (72%; OR = 0.06, *p* = 0.0073, 95% CI 0.01–0.47).

## Discussion

With a focus on high-burden disease at presentation, a low-dose Etoposide-Cisplatin regimen was introduced in the 1990s for both advanced GCT and TN [4, 5], defined here as the Em-EP regimen. Em-EP has been reported to reduce early deaths at less than 4 weeks in high-risk gestational TN (GTN) patients with a FIGO score ≥ 7 and even more so in those scoring ≥13 [5]. In this setting, an induction chemotherapy regimen administered at a lower dose than in standard regimens aims to reduce the tumour bulk more gradually, thereby minimising the risk of early treatment-related deaths, for example, from haemorrhage or rapid tumour lysis.

In our series, Em-EP appears to be an effective emergency chemotherapy regimen associated with an early favourable response in critically unwell patients presenting with advanced GCT or TN. Despite the presence of symptomatic disease, high-volume disease or organ failure, Em-EP allows for clinical stability to be achieved prior to embarking on a definitive chemotherapy regimen. In our experience, there are few situations when Em-EP cannot be administered, with each acute admission evaluated on an individual basis. Full organ support within a High Dependency or Intensive Care Unit may be required without delaying Em-EP administration. For example, concurrent antibiotics can be given alongside Em-EP to treat any co-existing sepsis and a profound transaminitis that has arisen from disease-related hepatic impairment can be monitored without prompting a treatment delay as long as the serum bilirubin remains within normal limits. There were 5 cases of neutropenic sepsis (5%) that occurred post Em-EP and prior to the first cycle of standard chemotherapy. Chemotherapy regimens with a febrile neutropenia rate at 10% or more could be offered prophylactic GCSF routinely. Given the context described within our cohort and the high risk for concomitant sepsis, we are working towards offering prophylactic GCSF to all patients at our ECTC who embark on Em-EP.

At our ECTC, Em-EP delivery has increased annually from 2013 onwards (Additional files 1 and 2). In particular, as a 24/7 National service, the vast majority of patients receive Em-EP within 24 h following presentation. We aim to specifically improve the clinical outcome for the high-risk patients that are directly referred to us. Within our 5-year analysis, early mortality after Em-EP administration remains low with 98% patients still alive at 4 weeks. There is a lack of published data on early outcomes in advanced GCT patients treated within the acute setting for symptomatic high-burden disease, with or without organ failure, either at low or conventional doses. For high-risk TN patients with a large disease burden, a study at our Centre by Alifrangis et al. [1] identified a low early death rate at 0.7% with upfront Em-EP compared to 7.2% for patients who proceeded with immediate conventional-dose EMA-CO chemotherapy. In our study, the TN subgroup recruited after 2012 differs from the cohort originally described by Alifrangis et al. who received low-dose chemotherapy up until 2010. The TN cohort serves as an important comparator and we demonstrate here that early mortality at 4 weeks is equivalent for both GCT and TN. Our findings suggest that Em-EP is safe and efficacious within our defined patient cohort where standard chemotherapy given on standard timelines and at a full dose could compromise clinical outcome due to unacceptable toxicity.

Interestingly, the extremely high overall cure rates of > 97% for male GCT [18], 85.6% for female GCT [19] and > 98% for GTN [22] are mostly accounted for by early detection and prompt treatment in early-stage disease. In contrast, the rarer and potentially lethal presentations for patients with symptomatic advanced disease described in this study are clearly associated with a more adverse clinical outcome. The 5-year overall survival rate stands at 48% only for male non-seminomatous GCT patients within a poor-risk IGCCCG category and most male GCT patients within our study presented acutely within this prognostic group. Our Em-EP study suggests that although patients with advanced TN can still fare well following an emergency presentation, patients with advanced GCT who present as an emergency harbour a less favourable clinical outcome particularly in IGCCCG poor-risk non-seminomatous disease for men. Female GCT patients were also more likely to develop treatment resistance and relapse following Em-EP and standard chemotherapy. Whether or not Em-EP administration independently identifies ultra high-risk patients with a very poor prognosis at presentation requires further evaluation in a large, multicentre prospective study.

As identified here within the acute setting, emergency presentation represents a well-recognised route to a cancer diagnosis in teenagers and young adults. So why are these young patients with GCT and TN presenting so late? Within our Em-EP study cohort, the disease had frequently progressed to an advanced stage requiring urgent chemotherapy, subsequently followed by an intensive and more complex treatment approach including at least one surgical procedure (Additional files 1 and 2), which in several cases failed to prevent disease relapse and subsequent death. Prospects in achieving a cure can therefore be diminished or extinguished with such late disease presentations, particularly when there is symptomatic disease, high-volume disease or impending organ failure. Hence, earlier cancer detection in teenagers and young adults would circumvent the adverse clinical outcomes described here.

Limitations to our study include the relatively low number of cases analysed, although the diseases described are rare when compared to other tumour types, hence why our analysis included both GCT and TN given that Em-EP represents a common chemotherapy of choice within the acute setting at our Institution. We further encountered a limited clinical follow-up for the period studied with the retrospective method utilised. Also, specific case details were occasionally found to missing due to the limited access to archival hand-written clinical notes subsequently replaced by electronic patient records during the study period.

Whilst GTN has a clearly-defined ‘high’ versus ‘low’ risk FIGO prognostic scoring for induction low-dose EP, a clearer prognostic classification for GCT patients presenting as an emergency should be sought based on a larger, multicentre-based prospective study. Moreover, it will be interesting to compare clinical outcome between GCT patients who receive Em-EP prior to definitive chemotherapy versus a matched cohort that do not. Other relevant areas for research include a translational substudy and an analysis on serum tumour marker kinetics, which demonstrated no clear correlation in our study but have been demonstrated in previous studies to predict clinical outcome in specific male GCT [16] and female GCT cohorts [7]. Furthermore, a more detailed safety evaluation on Em-EP with Common Terminology Criteria for Adverse Events (CTCAE) toxicity grading is warranted, as well as consideration for expanding the indications for Em-EP to other platinum-sensitive malignancies including advanced small-cell lung cancer.

## Conclusions

Our ECTC based at a designated cancer centre has an on-site Emergency Department and patients are referred to our 24/7 service for emergency cancer treatment such as the Em-EP regimen described in this paper. This strategy represents a comprehensive approach for delivering high-quality cancer care within the emergency setting. From this initial Em-EP study, we recommend that specialist units treating advanced GCT and TN in this context make Em-EP available 24/7 for patients with the high-risk clinical features defined here.

## Additional files

Additional file 1: **Figure S1.** Em-EP service delivery. Patients treated with Em-EP per annum. (PDF 45 kb)

Additional file 2: **Table S1.** Reasons for treatment delays (> 24 h) with Em-EP. **Table S2.** Surgical procedures in GCT patients who received Em-EP and conventional chemotherapy. **Table S3.** Causes of death within the Em-EP cohort. (DOC 20 kb)

## Data Availability

Please contact corresponding author for review of dataset spreadsheet.
