# Supplementary figures and images for: Emergency Etoposide-Cisplatin (Em-EP) for patients with germ cell tumours (GCT) and trophoblastic neoplasia (TN)
Source: BMC Cancer. 2019 Aug 5;19:770. doi: 10.1186/s12885-019-5968-7 (PMC6683367; doi:10.1186/s12885-019-5968-7)

Data Supplement

Supp Fig 1

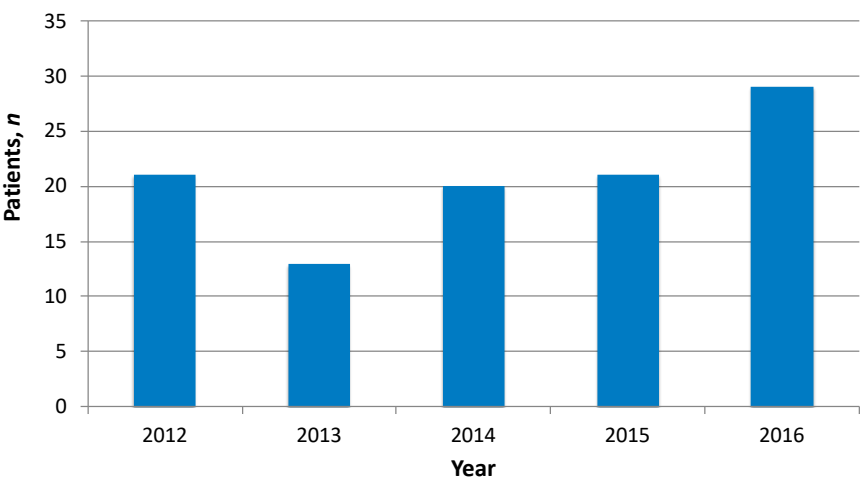

Supplement: Supplementary file 1 — Figure S1. Em-EP service delivery. Patients treated with Em-EP per annum. (PDF 45 kb) [file 12885_2019_5968_MOESM1_ESM.pdf]
