# Supplementary material for: Emergency Etoposide-Cisplatin (Em-EP) for patients with germ cell tumours (GCT) and trophoblastic neoplasia (TN)
Source: BMC Cancer. 2019 Aug 5;19:770. doi: 10.1186/s12885-019-5968-7 (PMC6683367; doi:10.1186/s12885-019-5968-7)
Supplement: Supplementary file 2 — Table S1. Reasons for treatment delays (> 24 h) with Em-EP. Table S2. Surgical procedures in GCT patients who received Em-EP and conventional chemotherapy. Table S3. Causes of death within the Em-EP cohort. (DOC 20 kb) [file 12885_2019_5968_MOESM2_ESM.doc]

**Table S1. Reasons for treatment delays (>24 hours) with Em-EP**. Although most patients were treated promptly, 4 patients were found to have started Em-EP more than 3 days after their initial diagnosis.

| Disease | Reasons for delay in Em-EP administration |
| --- | --- |
| GCT | A male GCT patient with a 7-day delay required an excision biopsy and histological diagnosis prior to initiating Em-EP.  A male GCT patient had a 14-day gap prior to Em-EP for a presumed GCT, with a significantly elevated AFP level, without an obvious primary tumour. |
| TN | A TN patient started Em-EP at 22 days following local ERPC because the hCG level initially fell spontaneously and subsequently reached a plateau, which triggered the decision to admit and treat with Em-EP.  A TN patient experienced a 10-day delay in Em-EP administration initially due to patient choice then complicated by chest sepsis requiring admission to a different hospital and clinical stabilisation. |

**Table S2. Surgical procedures in GCT patients who received Em-EP and conventional chemotherapy**. Despite this multimodality approach, more female (n=18, 58%) than male GCT patients (n=9, 41%) developed resistance and relapsed following Em-EP and standard chemotherapy. In contrast, only approximately one quarter (23%, *n*=12) patients with the highly chemosensitive TN relapsed following Em-EP, conventional chemotherapy and no surgery.

| Surgical procedure | Male GCT patients, n=14 (64%) | | Female GCT patients, n=21 (68%) | |
| --- | --- | --- | --- | --- |
| Description | Radical inguinal orchidectomy | n*=*6 (43%) | Debulking laparotomy | n*=*16 (76%)  n*=*5 (24%) including hysterectomy |
|  | Retroperitoneal lymph node dissection (RPLND) | n=7 (50%) | Residual extragonadal mass resection | n*=*3 (14%) |
|  | Resection involving post-chemotherapy residual masses | n=7 (50%), including video-assisted thoracoscopic surgery (VATS) in 1 (17%) | Thoracotomy | n*=*1 (5%) |
| Single procedure | n=11 (50%) | | n=17 (57%) | |
| Two procedures | n=3 (14%) | | n=4 (13%) | |

**Table S3. Causes of death within the Em-EP cohort.** Disease progression was observed in 9 patients (60%), sepsis in 2 patients (13%, 1 with neutropaenic sepsis) and a cause unknown in 4 patients (27%). Four patients (27%) had received chemotherapy within 30 days prior to their death and 11 patients (73%) were not on any systemic treatment within 30 days from their date of death. Amongst the 15 deaths, the serum tumour marker trend (hCG, AFP or LDH) before compared to immediately after Em-EP was rising in 7 patients (47%), falling in 7 patients (47%) and stable in 1 patient (7%).

| **Disease** | **Death details** |
| --- | --- |
| **GCT** | **Males**. For the 6 documented deaths observed at follow-up in males, 4 occurred in histologically-confirmed GCT cases, excluding patients with carcinoma of unknown primary or poorly-differentiated marker-secreting tumours as well as or lung primaries treated as GCT. Of the 4 confirmed GCT deaths (3 non-seminomas, 1 seminoma), all had Stage III disease. Documented causes of death included: disease progression (*n*=1, 25%) and neutropaenic sepsis (*n*=1, 25%). In 2 cases the causes of death at follow-up were unknown (50%). Two deaths occurred within the IGCCCG poor prognostic category, one of which had intracranial metastases at presentation and another had widespread liver metastases at presentation. One death was in an IGCCCG intermediate prognosis group patient who received Em-EP for relapsed disease and presented with a soft tissue mass around L2-4 resulting in leg weakness. Unfortunately, there was one death in a good prognosis group seminoma patient who received one cycle of BEP following Em-EP. He subsequently died without a fixed abode 8 days later of an unclear cause of death occurring early at less than 4 weeks from the initial presentation date.  **Females**. Amongst 10 documented deaths in female GCT, 8 (80%) were in Stage IV disease, 1 (10%) in Stage III disease and one in an unclassified patient (10%) with anaplastic histology. Documented causes of death included disease progression (*n=*7, 70%) and sepsis (*n=*1 10%) with the cause unknown in 2 patients (20%). A prognostic score is not clearly defined for female GCT patients, however 2 patients had intracranial metastases and 3 had liver metastases at presentation. None of these deaths were early deaths at less than 4 weeks from the date of first presentation. |
| **TN** | In terms of overall mortality, of the 4 documented deaths in the total TN cohort (8%), all were classified as choriocarcinomas: 1 gestational and 3 non-gestational cancer with unknown primaries. All deaths occurred in patients with poor prognostic Stage IV disease. The one GTN death occurred in a Stage IV gestational choriocarcinoma patient confirmed on histology and with widespread non-pulmonary visceral metastases (brain, spine, lung, liver, spleen, bladder and thyroid). The cause of death was disease progression. The remaining 3 deaths occurred in non-gestational choriocarcinomas of unknown primary with histology confirmed in 2 cases, with non-pulmonary visceral metastases occurring in liver, brain and leptomeninges. One of these patients required ITU admission for respiratory support with intubation, inotropic support as well as extracorporeal membrane oxygenation (ECMO) and subsequently died of respiratory failure due to sepsis (*n*=1). The remaining causes of death in this group were due to disease progression (*n*=2). |
